# Effects of AQP5 gene silencing on proliferation, migration and apoptosis of human glioma cells through regulating EGFR/ERK/ p38 MAPK signaling pathway

**DOI:** 10.18632/oncotarget.16461

**Published:** 2017-03-22

**Authors:** Jian Yang, Jian-Nan Zhang, Wei-Lin Chen, Gui-Song Wang, Qing Mao, Shan-Quan Li, Wen-Hao Xiong, Ying-Ying Lin, Jian-Wei Ge, Xiao-Xiong Li, Zhao Gu, Chun-Run Zhao

**Affiliations:** ^1^ Department of Neurosurgery, Renji Hospital, School of Medicine, Shanghai Jiaotong University, Shanghai 200127, P. R. China; ^2^ Operation Room, Shanghai Children's Medical Center, School of Medicine, Shanghai Jiaotong University, Shanghai 200127, P. R. China

**Keywords:** AQP5, U87-MG cells, U251 cells, LN229 cells, cell apoptosis

## Abstract

We investigated the effects of aquaporin 5 (*AQP5*) gene silencing on the proliferation, migration and apoptosis of human glioma cells through regulating the EGFR/ERK/p38MAPK signaling pathway. qRT-PCR was applied to examine the mRNA expressions of *AQP5* in five human glioma cell lines. U87-MG, U251 and LN229 cells were selected and assigned into blank, vector, *AQP5* siRNA and Flag*AQP5* groups. MTT assay was used to measure cell proliferation. Flow cytometry (FCM) with AnnexinV-FITC/PI double staining and PI staining were employed to analyze cell apoptosis and cell cycle respectively. Scratch test was used to detect cell migration. Western blotting was performed to determine the EGFR/ERK/p38 MAPK signaling pathway-related proteins. Results showed that the positive expression of *AQP5* in primary glioblastoma was associated with the tumor size and whether complete excision was performed. The mRNA expressions of *AQP5* in cell lines of U87-MG, U251 and LN229 were significantly higher than in U373 and T98G. The proliferation rates of U87-MG, U251 and LN229 cells in the *AQP5* siRNA group were lower than in the vector and blank groups. The apoptosis rate increased in the *AQP5* siRNA group compared with the vector group. Scratch test demonstrated that *AQP5* gene silencing could suppress cell migration. Compared with the vector and blank groups, the *AQP5* siRNA group showed decreased expressions of the ERK1/2, p38 MAPK, p-ERK1/2 and p-p38 MAPK proteins. *AQP5* gene silencing could inhibit the cell proliferation, reduce cell migration and promote the cell apoptosis of U87-MG, U251 and LN229 by suppressing EGFR/ERK/p38 MAPK signaling pathway.

## INTRODUCTION

Glioma, the most common malignant tumor of the central nervous system, is characterized by high morbidity, recurrence rate and mortality, and low cure rate [1]. World Health Organization guidelines classify glioma into grades I~IV according to the pattern of the tumor; the tumors in grades I-II are referred to low-grade gliomas (LGG) and those in grades III-IV are cited as high-grade gliomas (HGG) [2]. HGG, featured by high aggressiveness and poor prognosis, are by now the most common primary brain tumors occurred in adults [3, 4]. Patients with LGG have a relatively higher average survival time compared with those with HGG [5]. Surgery is still recognized as the first line therapy for glioma, though the tendency of glioma cells to infiltrate normal brain tissue makes it unachievable to conduct total resection, and glioma recurs in 90% of patients in the resection margin [6, 7]. Traditional treatment strategies typically combine tumor resection with radiotherapy and chemotherapy, but therapeutic effects are often unsatisfactory [8, 9]. Improvement in the outcome may depend on a better understanding of the scientific basis of glioma-genesis and the clinical application of such knowledge. Molecular mechanisms of the invasiveness and progression of glioma have been widely explored, but effective therapeutic target has not yet been found [10, 11].

Aquaporins (AQPs) are water channel proteins that have been recognized exist in virtually all living organisms, allowing water to move rapidly through the plasma membrane in almost cells [12]. Water-selective AQPs are involved in multiple biological functions, including neuro-excitation, cell migration, brain edema and trans-epithelial fluid transport. Modulators of AQP function suggest its clinical significance in neurology, oncology, nephrology and dermatological indications [13]. *Aquaporin5 (AQP5)*, one of subtypes of aquaporin family, is involved in regulation of mucin expression and secretion as well as fluid secretion of airway submucosal gland [14]. High *AQP5* expression in colon, breast and pancreatic cancer cells affects the invasion, proliferation and metastasis of the tumor cells [15, 16, 17]. In addition, *AQP5* gene silencing inhibits the proliferation of endometrial glandular epithelial cells notably [18], suggesting that altered *AQP5* expression plays a crucial role in tumor progression. Up-regulation of *Aquaporin1 (AQP1)* gene has been reported to promote the invasiveness of glioma cells [19, 20]. However, the role of *AQP5* gene expression in glioma has not been studied. In addition, epidermal growth factor receptor (EGFR) and mitogen-activated protein kinase (MAPK) have been reported to promote tumor proliferation, migration and invasion [21–23]. In this study, we investigated how *AQP5* gene silencing might influence the proliferation and apoptosis of human glioma cells and the involvement of the EGFR/extracellular signal-regulated kinase (ERK)/MAPK pathway to provide a new direction for the treatment of glioma.

## RESULTS

### Comparisons of the *AQP5* expression between primary glioblastoma and normal brain tissues

**Figure 1:**
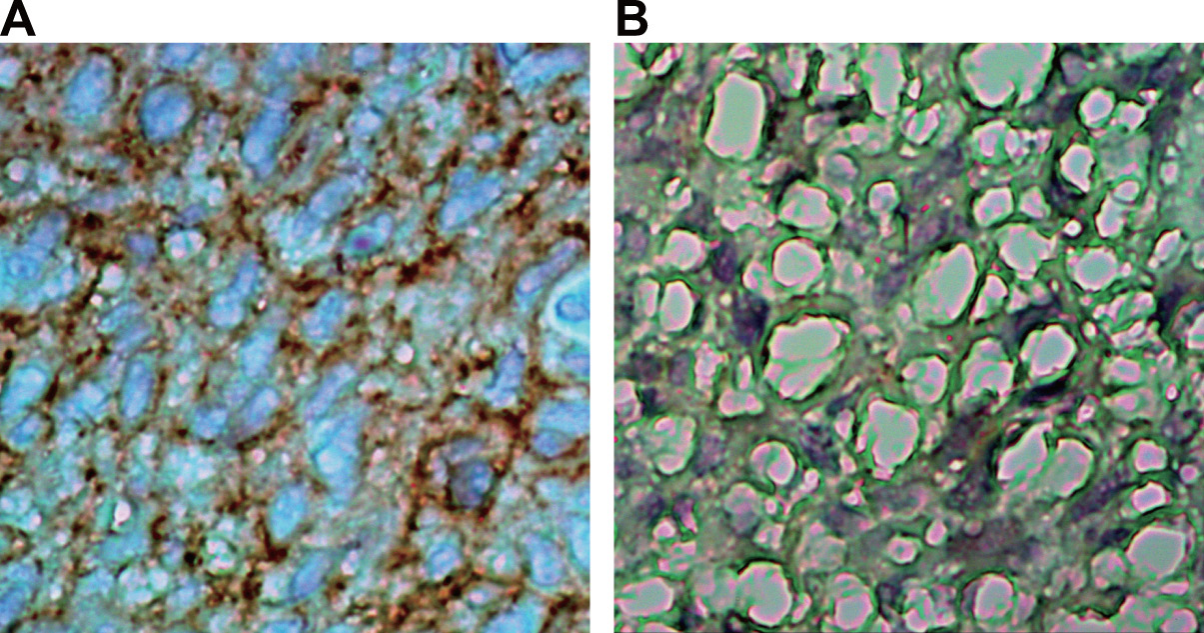
Comparisons of *AQP5* expressions in primary glioblastoma and normal brain tissue detected by IHC (× 400) (**A**) primary glioblastoma tissue; (**B**) normal brain tissue.

As shown in Figure 1. Compared with the normal brain tissue, the expressions of *AQP5* significantly increased in primary glioblastoma, and distributed both in cytoplasm and nuclei. These indicated that *AQP5* over expressed in primary glioblastoma.

### Association between the *AQP5* expression and clinicopathological characteristics of patients with primary glioblastoma

As shown in Table 1, the positive expression of *AQP5* in primary glioblastoma was associated with the tumor size and whether complete excision was performed (*P* < 0.05). The larger diameter and partial excision were accompanied with higher positive expression of *AQP5*. There was no association between the positive expression of *AQP5* and age, gender, KPS score and tumor location (*P* > 0.05).

**Table 1:** Association between the *AQP5* expression and clinicopathological characteristics of patients with primary glioblastoma

Clinicopathological characteristic		Case	Positive expression (case)	Positive rate (%)	χ^2^	*P*
Gender	Male	10	8	80.0	2.222	0.136
	Female	10	10	100.0		
Age(year)	> 50	14	12	85.7	0.952	0.329
	< = 50	6	6	100.0		
KPS score	> 70	9	9	100.0	1.818	0.178
	< = 70	11	9	81.8		
Tumor size	> 3 cm	15	15	100.0	6.667	0.010
	< = 3 cm	5	3	60.0		
Tumor location	Frontal lobe	6	4	66.7	5.185	0.745
	Temporal lobe	11	11	100.0		
	Other parts	3	3	100.0		
Tumor excision	Complete excision	4	2	50.0	8.889	0.003
	Partial excision	16	16	100.0		

Note: Chi-square test was used for analysis.

### Screening of cell lines with *AQP5* overexpression

**Figure 2:**
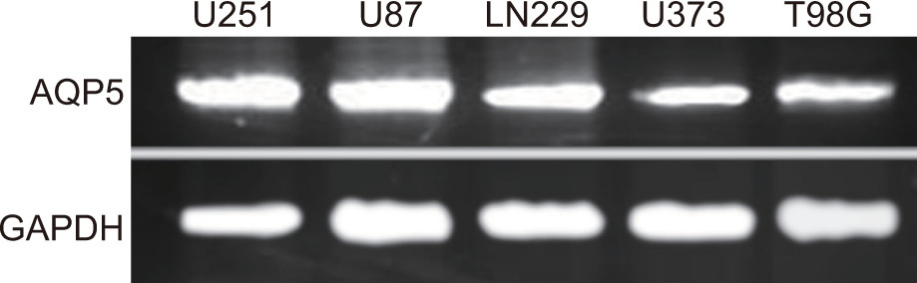
The mRNA expressions in U87-MG, U251, U373, T98G and LN229 cells detected by qRT-PCR

qRT-PCR was applied to examine the mRNA expressions of *AQP5* in cell lines of U87-MG, U251, U373, T98G and LN229. As shown in Figure 2, the mRNA expressions of *AQP5* in U87-MG, U251 and LN229 were significantly higher than in U373 and T98G. Therefore, U87-MG, U251 and LN229 were chosen in this study for further experiments.

### Transfection efficiency of AQP5 overexpression plasmid and the AQP5 siRNA plasmid

**Figure 3:**
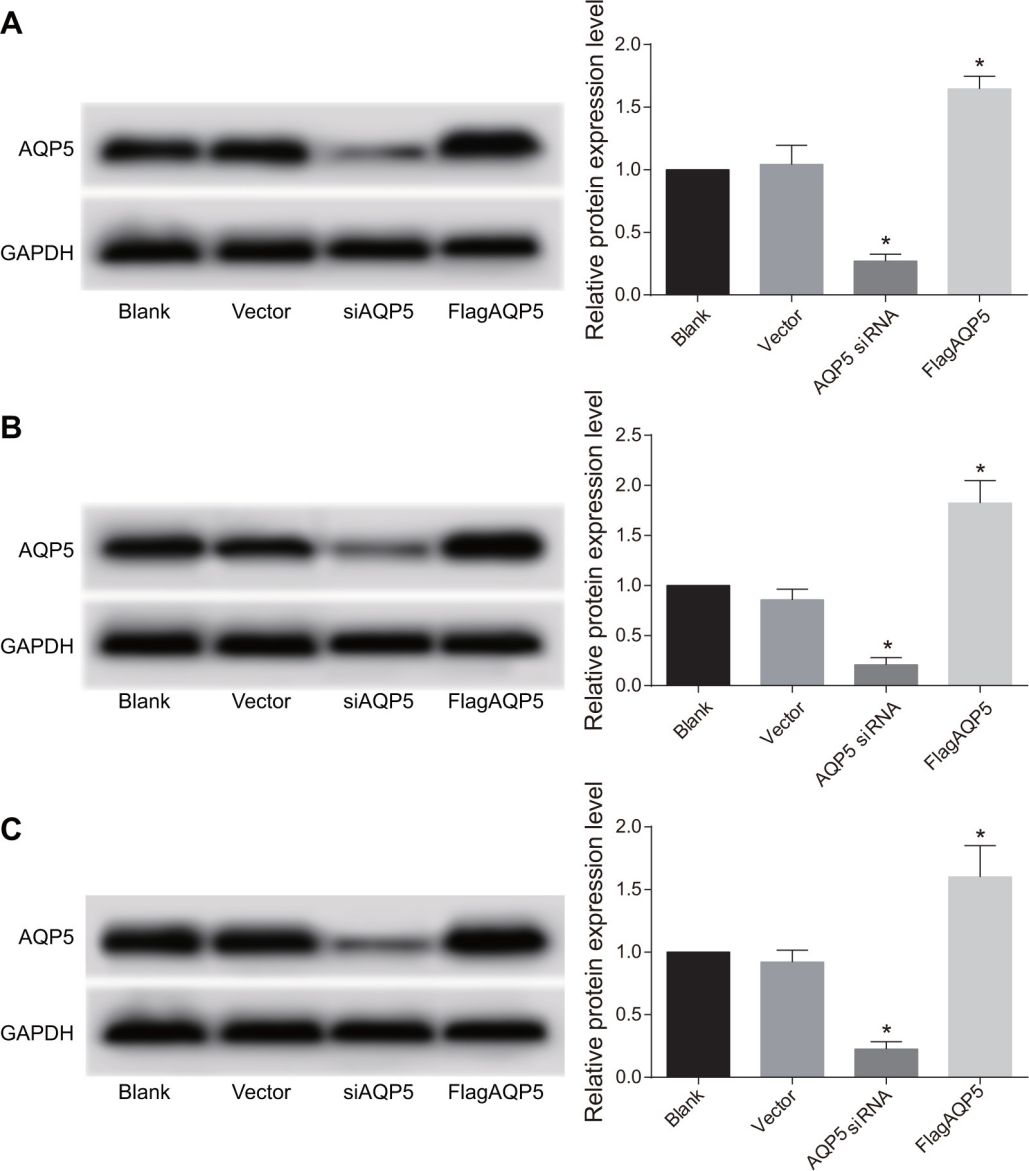
Transfection efficiency of *AQP5* siRNA and Flag*AQP5* in U87-MG, U251 and LN229 cells detected by Western Blotting (**A**) the expressions of *AQP5* in U87-MG among four groups; (**B**) the expressions of *AQP5* in U251 among four groups; (**C**) the expressions of *AQP5* in LN229 among four groups; one-way analysis of variance (ANOVA) was used for analysis and the experiment was repeated for three times; *P < 0.05 compared to the vector and blank groups.

U87-MG, U251 and LN229 were transfected with *AQP5* siRNA and Flag*AQP5*, and transfection efficiency of overexpression plasmid and *AQP5* siRNA plasmid were detected by Western Blotting (Figure 3). Compared with the vector group, in U87-MG, U251 and LN229 cells it could be found that *AQP5* gene silencing reduced *AQP5* protein levels by more than 75% and transfection efficiency of *AQP5* siRNA reached more than 75% (*P* < 0.05). In the Flag*AQP5* group, the expressions of AQP5 in U87-MG, U251 and LN229 cells at least doubled (*P* < 0.05).

### Effect of AQP5 gene silencing on the proliferation of U87-MG, U251 and LN229 cells

**Figure 4:**
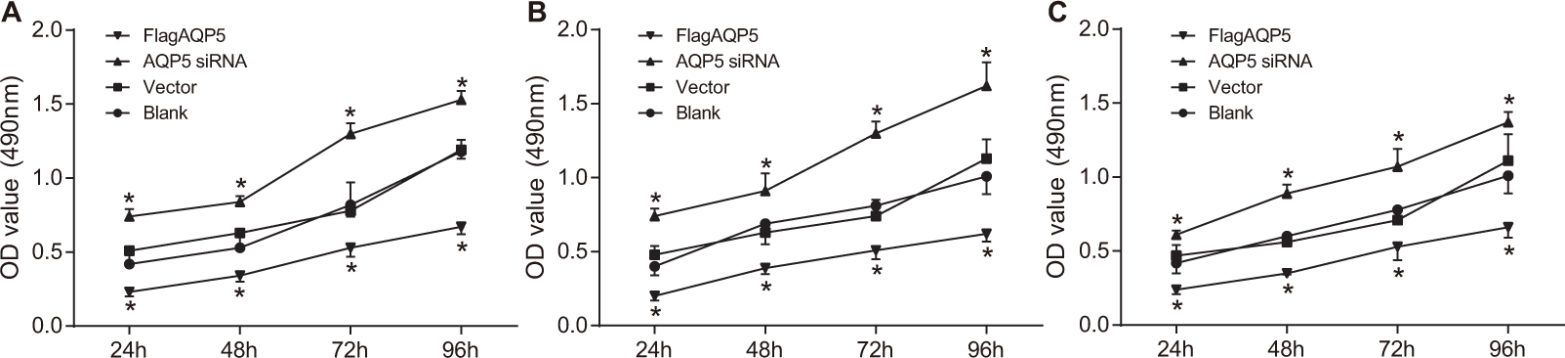
Effect of *AQP5* gene silencing on the proliferation of U87-MG, U251 and LN229 at four time points (**A**) the proliferation of U87-MG in four groups at 24 h, 48 h, 72 h and 96 h after transfection; (**B**) the proliferation of U251 in four groups at 24 h, 48 h, 72 h and 96 h after transfection; (**C**) the proliferation of LN229 in four groups at 24 h, 48 h, 72 h and 96 h after transfection; repeated measures analysis of variance was used for comparisons and the experiment was repeated for three times; *P < 0.05 compared to the vector and blank groups at the same time point.

MTT assay was used to determine the effect of *AQP5* gene silencing and *AQP5* overexpression on the proliferation of U87-MG, U251 and LN229 cells at time points of 24 h, 48 h, 72 h and 96 h after transfection (Figure 4). With time increasing, the proliferation rates of U87-MG, U251 and LN229 cells in each group were significantly increased (*P* < 0.05). At each time point, there was no significant difference in the proliferation rates between the vector group and the blank group (*P* > 0.05), however, the proliferation rates in the *AQP5* siRNA group was significantly decreased compared with those in the vector and blank groups (*P* < 0.05). The proliferation rates in the Flag*AQP5* group were significantly higher than other groups (*P* < 0.05).

### Effect of AQP5 gene silencing on cell cycle distribution of U87-MG, U251 and LN229 cells

Cell cycle distribution of U87-MG, U251 and LN229 cells was analyzed at 24 h after transfection. As shown in Supplementary Figure 1, no difference in cell cycle distribution was found between the vector and blank groups. However, in the *AQP5* siRNA group, the proportion of cells in the G0-G1 phase were significantly increased while the proportion of cells in S phase were significantly decreased, and there was no significant difference in percentages of cells in G0-G1 and S phase between the Flag*AQP5* group with the vector group. As shown in Table 2, *AQP5* gene silencing increased the proportion of cells in the G0-G1 phase and decreased the proportion of cells in S phase (all *P* < 0.05).

**Table 2:** Cell cycle distribution of U87-MG, U251 and LN229 cells among the blank, vector, *AQP5* siRNA and Flag*AQP5* groups

Cell line	Group	Cell cycle distribution (%)		
		G0-G1 phase	G2-M phase	S phase
U87-MG	Blank	57.74 ± 6.04	9.67 ± 1.43	32.59 ± 7.41
	Vector	58.94 ± 6.36	9.82 ± 1.21	31.24 ± 7.51
	*AQP5* siRNA	69.34 ± 3.73*	9.61 ± 0.95	21.05 ± 4.62*
	Flag*AQP5*	56.36 ± 3.10	9.53 ± 1.93	34.11 ± 4.82
U251	Blank	58.96 ± 2.58	10.20 ± 0.82	30.82 ± 2.14
	Vector	58.84 ± 6.11	9.01 ± 0.34	32.15 ± 6.12
	*AQP5* siRNA	68.33 ± 4.48*	9.66 ± 1.25	22.01 ± 3.43*
	Flag*AQP5*	57.26 ± 4.76	10.33 ± 0.45	32.42 ± 5.17
LN229	Blank	64.47 ± 2.97	9.50 ± 3.13	26.03 ± 3.83
	Vector	63.03 ± 3.92	10.53 ± 1.97	26.44 ± 2.36
	*AQP5* siRNA	70.70 ± 4.02*	8.59 ± 1.56	20.70 ± 4.37*
	Flag*AQP5*	60.85 ± 3.48	9.68 ± 1.36	29.48 ± 2.49

Note: *P < 0.05 compared with the vector and blank groups; one-way analysis of variance (ANOVA) was used for analysis and the experiment was repeated for three times.

### Effect of *AQP5* gene silencing on the apoptosis of U87-MG, U251 and LN229 cells

**Figure 5:**
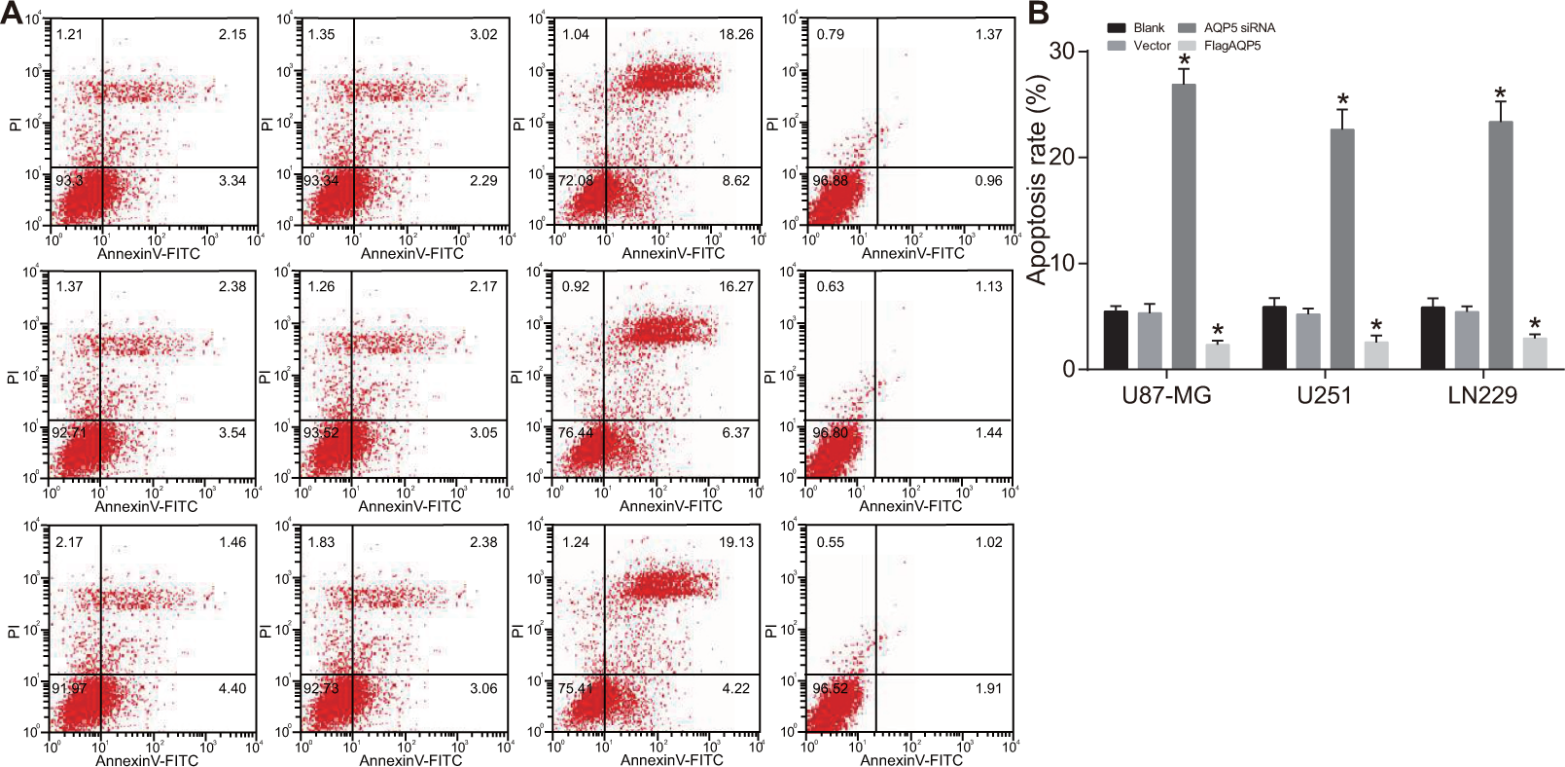
Effect of *AQP5* gene silencing on the cell apoptosis of U87-MG, U251 and LN229 (**A**) representative dot plots of AnnexinV-FITC /PI in U87-MG, U251 and LN229 cells in each group; (**B**) the apoptosis rate of U87-MG, U251 and LN229 cells in each group; one-way analysis of variance (ANOVA) was used for analysis and the experiment was repeated for three times; *P < 0.05 compared to the vector and blank groups.

There was no difference in cell apoptosis rates between the vector and blank groups (both *P* > 0.05) (Figure 5A, 5B). However, the apoptosis rate of U87-MG, U251 and LN229 increased significantly in the *AQP5* siRNA group while decreased significantly in Flag*AQP5* group compared to the vector group (*P* < 0.05). These results indicated that *AQP5* gene silencing increased apoptosis in U87-MG, U251 and LN229 cells.

### Effect of *AQP5* gene silencing on the migration of U87-MG, U251 and LN229 cells

**Figure 6:**
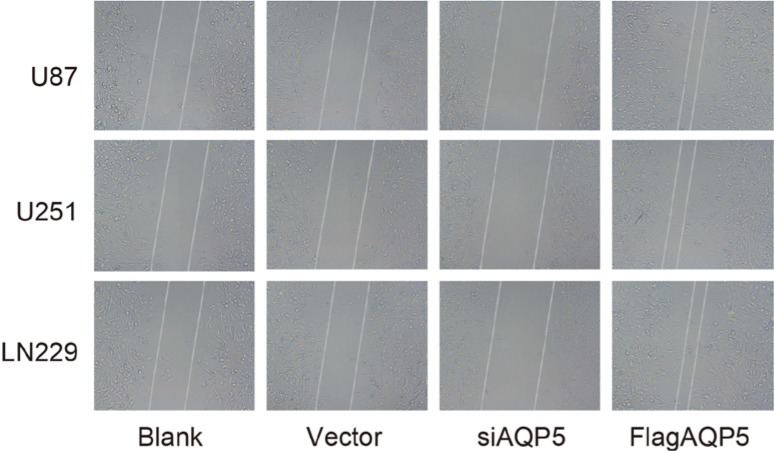
Effect of *AQP5* gene silencing on the cell migration of U87-MG, U251 and LN229

As shown in Figure 6. There was no significant difference in migration ability of U87-MG, U251 and LN229 cells between the vector and blank groups. In the *AQP5* siRNA group, the migration abilities were significantly weaker than in the vector and blank groups, while in the Flag*AQP5* group, the abilities were stronger than in the vector and blank groups. The findings showed that *AQP5* gene silencing reduced migration ability of U87-MG, U251 and LN229 cells.

### Effect of *AQP5* gene silencing on the activation of EGFR/ERK/p38 MAPK signaling pathway

**Figure 7:**
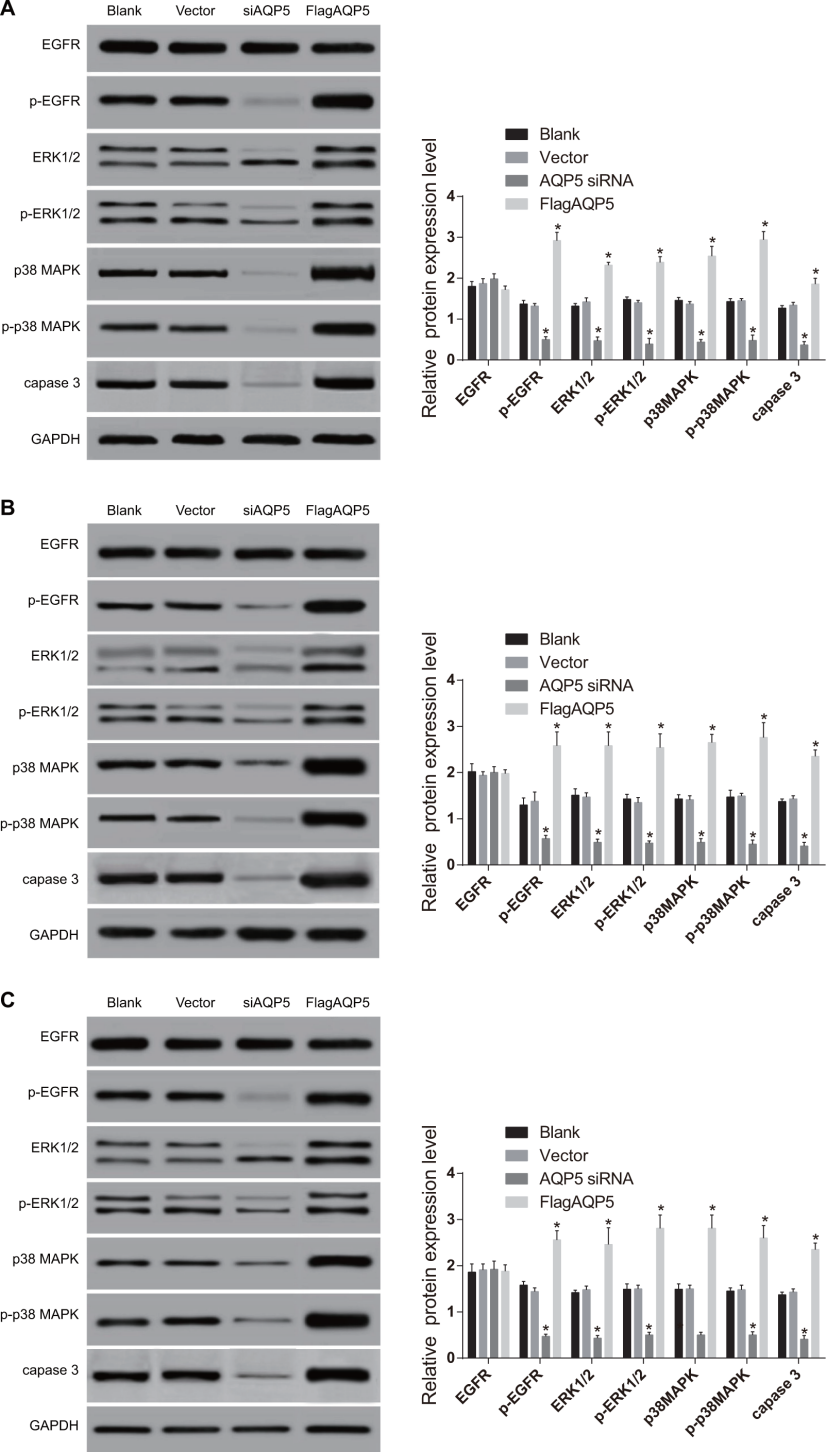
Effect of *AQP5* gene silencing on the expressions of EGFR/ERK/p38 MAPK signaling pathway-related proteins in U251, U87-MG, and LN229 cells detected by Western Blotting (**A**) U87-MG; (**B**) U251; (**C**) LN229; one-way analysis of variance (ANOVA) was used for analysis and the experiment was repeated for three times; *P < 0.05 compared to the vector and blank groups.

As shown in Figure 7, the expressions of EGFR/ERK/p38 MAPK signaling pathway-related proteins in U87-MG, U251 and LN229 cells showed no significant difference between the vector and blank groups (all *P* < 0.05). Compared with the vector and blank groups, the *AQP5* siRNA group showed significantly decreased expressions of the p-EGFR, p-capase 3, ERK1/2, p-ERK1/2, p38 MAPK and p-p38 MAPK proteins in U87-MG, U251 and LN229 cells (all *P* < 0.05). While there was no difference in the protein expressions of EGFR among four groups (all *P* > 0.05). On the contrary, the expressions of p-EGFR, ERK1/2, p-ERK1/2, p38 MAPK, p-p38 MAPK and capase 3 proteins in U87-MG, U251 and LN229 cells of the Flag*AQP5* group were significantly higher than in the vector group and blank group. The results indicated that *AQP5* gene silencing could inhibit the activation of the EGFR/ERK/p38 MAPK signaling pathway.

## DISCUSSION

In this study, we investigated the effects of *AQP5* gene silencing on the proliferation, migration and apoptosis of U87-MG and U251 glioma cells through regulating EGFR/ERK/p38 MAPK signaling pathway. Our findings have demonstrated that *AQP5* gene silencing inhibited the proliferation, suppressed migration and promoted the apoptosis of U87-MG, U251 and LN229 glioma cells, indicating that *AQP5* may be a promising target for novel drug development for glioma.

Small interfering RNA (siRNA), an RNAi application, has been widely used for gene silencing, including in glioma [25–27]. Here, we found that siRNA-mediated *AQP5* gene silencing inhibited the proliferation of U87-MG, U251 and LN229 glioma cells. This inhibition effect likely resulted from *AQP5* gene silencing-induced cell cycle arrest in the G0-G1 phase. Meanwhile, the ability of *AQP5* in promoting cell proliferation may be dependent on the phosphorylation of a cAMP-protein kinase (PKA) consensus site that is located in a cytoplasmic loop of *AQP5*, and phosphorylation of the PKA consensus site is preferentially in tumors [28]. Therefore, blocking phosphorylation of the site might have therapeutic benefits. *AQP5* have been found expressed highly in primary human glioma cells compared with normal tissue, this is in accordance with the study that reported *AQPs* (such as *AQP1*, *AQP5* and *AQP8*) have been observed in high-grade tumors of various tissues [29, 30]. It is confirmed that high expression of *AQP5* was associated with tumor growth, invasion and metastasis in non-small cell lung cancer [31]. Yang et al. found that *AQP5* expression was increased in borderline ovarian tumors and malignant tumors compared to benign and normal ovarian tumors, and that *AQP5* expression was correlated with lymph node metastasis [32]. Together, these results suggest that high *AQP5* expression promotes tumor cell proliferation, which agrees with the suppression of tumor cell proliferation observed here after *AQP5* gene silencing.

In this study, scratch test showed significantly enhanced migration in Flag*AQP5* cells but down-regulation of *AQP5* in cells by *AQP5* gene silencing showed significantly decreased migration. This result provides evidence that there is a potential increased metastatic ability in *AQP5* over expressed cells and the effect could be weaken by suppressing *AQP5* gene. Tumor metastasis involves a series of complex host–tumor interactions and tumor cells interacted with the basal membrane believed to include cell attachment, matrix dissolution, and migration [33, 34]. One mechanism may considered that *AQPs* facilitated water permeability in cell protrusions that enhances their formation and accelerate the rate of cell migration [35, 36]. The study reported by Zhang et al. also showed notably increased water permeability in *AQP5* over expressed cells may be enhanced metastases and invasiveness of these cells [37].

Our study also showed that *AQP5* gene silencing could increase the apoptosis rate of U87-MG, U251 and LN229 cells. Apoptosis, controlled by gene expression, can help the body adapt to different environmental conditions [38]. The anti-tumor effects of many cancer drugs depend on the induction of apoptosis, and both pro-apoptotic and anti-apoptotic proteins play important roles in this process [39]. *AQP* gene silencing reduces levels of the apoptosis inhibiting proteins c-IAP1, c-IAP2, Livin and X-linked inhibitor of apoptosis protein (XIAP) [40]. Proteins c-IAP1 and c-IAP2 directly inhibit the activity of Caspase-3, Caspase-7 and Caspase-9, thereby inhibiting cell apoptosis [41]. Additionally, XIAP blocks and Livin inhibits could induce cell apoptosis by inhibiting Caspase and activating the TAK1/JNK1 pathways [42, 43]. *AQP5* gene silencing might increase apoptosis rates by reducing the levels of these apoptosis-inhibiting proteins.

It was found in the study that *AQP5* gene silencing could inhibit the EGFR/ERK/p38 MAPK signaling pathway. This result was consistent with the research by Zhang et al. that in *AQP5* highly expressed cells, EGFR phosphorylation was enhanced and the ERK and MAPK signaling pathway was activated, while deletion of *AQP5* decreased the activity of the EGFR/ERK/p38 MAPK pathway [44]. The EGFR/ERK/p38 MAPK pathway plays a crucial role in signal transduction in tumors, promoting tumor growth and migration at the transcriptional level [45, 46]. *AQP5* mediates proliferation and migration of tumor cells through the EGFR/ERK/p38 MAPK signaling pathway [47]. MAPK plays a significant role in the differentiation, proliferation and apoptosis of various cells, and ERK as one of three members of MAPK is mainly responsible for proliferative responses [48, 49]. Glioma cells may over-express EGFR, which is a tyrosine kinase receptor, resulting in cell proliferation and invasion with downstream effects [50]. VEGF is an important angiogenic factor for glioma, whose expression requires the activation of ERK/MAPK pathway [51]. ERK/MAPK signaling pathway is involved in epithelial-mesenchymal transition (EMT) transformation in SF767 glioma cells, promoting invasive tumor growth [52]. It is reported that *AQP1* plays an important role in water permeability and ultrafiltration, regulating endothelial permeability and angiogenesis [53], which may be further involved in EMT. We then boldly inferred that *AQP5* must function in a similar way as *AQP1* does. Therefore, we supposed that the effects of *AQP5* gene silencing on the proliferation, migration and apoptosis of U87-MG, U251 and LN229 cells might be due to its suppression to the activity of EGFR/ERK/p38 MAPK signal pathways.

In summarize, we demonstrated that *AQP5* gene silencing could inhibit the proliferation, reduce the migration and promote the apoptosis of human glioma cells by suppressing EGFR/ERK/p38 MAPK signaling pathway. These findings might be beneficial for the molecular mechanism underlying tumor cell proliferation, migration and apoptosis in human glioma. Nevertheless, further study for the role of *AQP5* on glioblastoma stem cells is still required to fully elucidate that if *AQP5* is associated with differentiation of glioma stem cells and whether it could be a potential therapeutic target in glioma.

## MATERIALS AND METHODS

### Ethical statement

This study was approved by the Clinical Experiment Ethical Committee of Renji Hospital, School of Medicine, Shanghai Jiaotong University and all participants signed a document of informed consent.

### Sample selection

From January 2014 to January 2015, twenty patients (age from 25~65 years) from Renji Hospital, School of Medicine, Shanghai Jiaotong University with primary human glioma no more than three months were recruited, including 10 males 10 females. The average age was (51.63 ± 11.62) years, there were 14 cases of age > 50 years and 6 cases of age < = 50 years. The postoperative Karnofsky Performance Scale (KPS) score was 50~100 points with an average score of (72.70 ± 8.44), there were 11 cases of > 70 points and 9 cases of < = 70 points. The diameter of human glioma tissue was 2~5 cm with an average value (3.91 ± 1.01). There were 6 cases of tumor located in frontal lobe, 11 cases in temporal lobe and 3 cases in other parts; 4 cases of complete excision and 16 cases of partial excision. Individuals (age from 28~60) with normal brain tissue were selected as control in the same period, including 10 males and 10 females. The samples of human glioma and normal brain tissue were taken from surgical resection. All subjects underwent radiotherapy and chemotherapy after the surgery.

### Immunohistochemistry (IHC)

Samples were fixed in 10% formaldehyde, embedded in paraffin and 5 μm sections were prepared. Sections were placed at room temperature for 60 min and then sections were immersed in xylene for dewaxing. Ethanol was used to hydration, afterwards, samples were blocked in 5% bovine serum albumin (BSA) at room temperature for 20 min. Sections were incubated in AQP5 primary antibody followed by placing at room temperature for 1 h, and then secondary antibody was added. After washing and the visualization with diaminobenzidine (DAB), sections were counterstained with hematoxylin for 2 min and differentiated by hydrochloric acid alcohol. Then sections were dehydrated, cleared, mounted and microscopy was performed. Images were acquired under the microscope (Olympus Corporation, Japan) and the intensity of the proteins were analyzed by counting the number of positive cells. Randomly selected 10 fields for view (× 400) and the criteria for results was: negative (−): no brown staining or positive cells < 10%; positive (+), positive cells > = 10%.

### Cell culture and treatment

Human glioma cell lines U87-MG, U251, U373, T98G and LN229 were purchased from Shanghai Institutes for Biological Sciences, Chinese Academy of Science (Shanghai, China). Cells were cultured in 10% Dulbecco's modified eagle medium (DMEM, pH = 7.2, Gibco Company, Grand Island, NY, USA) containing 10% fetal bovine serum (FBS) in an incubator at 37°C with 5% CO_2_. Cell growth and morphology were examined every day and medium was replaced every other day. Serial sub-cultivation was performed when cell density reached 80~85% and subsequent experiments were conducted 12 hours after cell adherence. Doubling time of human glioma cells U87-MG, U251, U373, T98G and LN229 were 32.45 ± 0.52 h, 36.45 ± 0.46 h, 32.45 ± 0.52 h, 36.45 ± 0.46 h and 30.96 ± 0.29 h, respectively. All cell lines were free from mycoplasma contamination (MycoAlert Mycoplasma Detection Kit, Sigma-Aldrich Chemical Company, St Louis MO, USA) in the cultivation.

### Quantitative real-time polymerase chain reaction (qRT-PCR)

Human glioma cells U87-MG, U251, U373, T98G and LN229 were collected in the 1.5 mL EP tube respectively, each tube with addition of 1 mL Trizol was followed by placing at room temperature for 10 min. Then 200 μL chloroform was added accompanied with shaking tubes violently for 10 s. After placing at room temperature for 5 min, cells were centrifuged at 12, 000 g at 4°C for 5 min, the supernatant was transferred to another tube subsequently for the next step. Then 200 μL isopropanol was added, and cells were placed at 4°C for 10 min and centrifuged at 12, 000 g at 4°C for 10 min. The pellets were washed by 1 mL 75% ethanol and followed by centrifugation at 12, 000 g at 4°C for 10 min. At last, the pellets were dissolved in diethylpyrocarbonate (DEPC) -treated water for 15 min. The concentration of RNA was calculated by the absorbance of RNA. Complementary DNA (cDNA) was synthesized from RNA samples (2 μg each sample). The reverse transcriptase reaction was performed: 3 μL of anchored oligo (dT) primers was added, DEPC-treated water was used to adjust total volume at 13 μL. Then heated at 5 min for 70°C (denaturation). After quick chilling on ice, the following were added (total 30 μL): 6 μL 5 × M-MLV buffer, 2 μL deoxyribonucleoside triphosphate (dNTP), 0.5 μL RNase inhibitor, 1.5 μL Moloney murine leukemia virus- reverse transcriptase (MMLV-RT) and DEPC-treated water. The condition of reverse transcription was: 42°C for 60 min, 95°C for 10 min, −40°C after termination. qRT-PCR was conducted using the PCR kit (A3500, Promega Corp., Madison, Wisconsin, USA) and the total volume of PCR reaction included 20 μL: 1 μL cDNA, 1 μL upstream primer, 1 μL downstream primer, 10 μL mixture and 8 μL DEPC-treated water. Forty cycles were performed under the conditions: pre-denaturation at 95°C for 10 min; denaturation at 94°C for 30 s, annealing at 72°C for 1 min, and extension at 72°C for 5 min. Then re-extension at 72°C for 5 min. The real-time PCR products were analyzed by 70 V agarose gel electrophoresis for 25 min and pictured under the UV light. GAPDH was amplified as the internal control and primer sequences were: upstream: 5′ -ATCACTGCCACCCAGAAGAC-3′, downstream: 5′- AGATCCACGACGGACACATT-3′. *AQP5* primer sequences were: upstream: 5′ -CTATGAG TCCGAGGAGGATT-3′, downstream: 5′- GCTTCGCTG TCATCTGTT-3′ [24].

### *AQP5* siRNA design and vector construction

*AQP5* specific small interfering RNA (*AQP5* siRNA) sequences were 5′-CGGUGGUCAUGAAUCGG UUTT-3′ for the sense strand and 5′-AACCGAUUCAUGA CCACCGCA-3′ for the anti-sense strand. The sense strand sequences for negative control siRNA were 5′-UUCU CCGAACGUGUCACGUTT-3′ and the anti-sense strand were 5′-ACGUGACACGUUCGGAGAATT-3′.

### Cell transfection

Human glioma cells U87-MG, U251 and LN229 were assigned into four groups respectively: the blank group (no transfection), the vector group (transfected with empty plasmid), the Flag*AQP5* group (transfected with overexpression plasmid) and the *AQP5* siRNA group (transfected with *AQP5* siRNA plasmid). Cells were seeded in a 6-well plate at a density of 10^5^ cell/well and each well containing 2 mL of DMEM with 10% FBS. Cells were then cultured for 12 h, and transfection was performed when cells reached density of 80%. Cells were subjected to starvation in 800 μL/well serum- and antibody-free DMEM medium before transfection. Mixed medium of different experimental plasmid and transfection reagent was prepared. Six sterile 1.5 mL EP tubes were selected: For tubes 1, 3 and 5: 800 ng of overexpression plasmid Flag*AQP5*, *AQP5* siRNA plasmid and empty plasmid were added to 50 μL of serum- and antibody-free DMEM medium. For tubes 2, 4 and 6: 2.5 μL of GeneJet^™^ II DNA extracorporeal transfection reagent (SIGNAGEN LABORATORIES LLC., Madrid, Spain) was added to 50 μL of serum- and antibody-free DMEM medium. Tubes 1 and 2, tubes 3 and 4 as well as tubes 5 and 6 were mixed respectively, and preserved at room temperature for 20 min. Then the mixed medium was transferred into the 6-well plate at 100 μL per well followed by 6 h of incubation at 37°C with 5% CO_2_, and another 24 h of incubation was performed with addition of 100 μL of complete culture medium to each well. After that, cells were collected for further use.

### 3-(4, 5-Dimethylthiazol-2-yl)-2, 5-diphenyltetrazolium bromide (MTT) assay

U87-MG, U251 and LN229 cell lines in the logarithmic phase were independently assigned into the blank, vector, Flag*AQP5* and *AQP5* siRNA groups, and after trypsin digestion, cell suspension was prepared. Cells in each group were seeded in a 96-well plate at 10^3^ cell/well. Four duplicate wells were seeded for each group. Four hour prior to cell collection, 10 μL of MTT solution (5 μg/μL) was added to each well. Medium was then removed and replaced with 100 μL of dimethyl sulfoxide (DMSO) in each well followed by incubation for 10 min protected from light. An ultraviolet spectrophotometer (Thermo Fisher Scientific Inc., USA) was used to detect absorbance of each well at the 490 nm wavelength.

### Flow cytometry with AnnexinV-FITC/PI staining

AnnexinV-FITC/PI methods were used to separate early apoptotic cells from late apoptotic cells. U87-MG, U251 and LN229 cell lines in the logarithmic phase were independently assigned into the blank, vector, Flag*AQP5* and *AQP5* siRNA groups. Cells were treated with cisplatin (CDDP, 4 μM/L) 24 h before transfection. An AnnexinV-FITC/PI kit (Becton, Dickinson and Company, New Jersey, USA) was used to stain cells for 15 min protected from light after transfection for 24 h. Flow cytometry and fluorescence activated cell sorting (FACS) (Becton, Dickinson and Company, New Jersey, USA) were applied to detect cell apoptosis immediately after incubation. The experiment was repeated for three times in each group.

### Flow cytometry with PI staining

U87-MG, U251 and LN229 cell lines in the logarithmic phase were independently assigned into the blank, vector, Flag*AQP5* and *AQP5* siRNA groups. Cells were seeded in a 6-well plate for 24 h of transfection. Digestion was performed using 0.25% trypsin, and the supernatant was discarded after 5 min of centrifugation at 3000 g at 4°C. Cells were washed three times with phosphate buffer (PBS), and pre-cooled 75% ethanol was then applied to fix cells overnight. Ethanol was discarded after 5 min of centrifugation at 3000 g at 4°C, and cell concentration was adjusted to 10^5^ cell/mL. Cell suspension (1 mL) was extracted for PI staining with a 20 min of incubation protected from light at 37°C. Flow cytometry and ModFit Software (Verity Software House Inc., Topsham, ME, USA) were used to detect cell cycle and to calculate percentages of the total cells in each stage. The experiment was repeated for three times in each group.

### Scratch test

U87-MG, U251 and LN229 cell lines in the logarithmic phase were independently assigned into the blank, vector, Flag*AQP5* and *AQP5* siRNA groups. Cells were plated into a 6-well plate at a density of 5 × 10^5^ cell/mL. When the density reached to 90%, cells were cultured in serum-free medium and starved for 24 h, and when the density reached to 100%, using a sterile 200 μL pipette tips to draw a line softly and quickly in monolayer cultures of cells. Then suspended cells were washed away by PBS, and cells were cultured in serum-free medium continually. After incubation for 24 h, migration of the cells was photographed.

### Western blotting

Supernatant was discarded and U87-MG, U251 and LN229 cells were collected and independently assigned into the blank, vector, Flag*AQP5* and *AQP5* siRNA groups, followed by washing three times with PBS. Cells were gently scraped and transferred to 1.5 mL EP tubes, and the supernatant was discarded after 5 min of centrifugation at 3000 g at 4°C. Cells were lysed in radioimmunoprecipitation assay (RIPA) buffer on ice for 40 min, followed by 10 min of centrifugation at 12000 g at 4°C. The supernatant was extracted and a bicinchoninic acid (BCA) kit (Beyotime Biotechnology Co., Shanghai, China) was used to determine the protein concentration of U87-MG, U251 and LN229 cells. Total protein (50 μg) of U87-MG, U251 and LN229 cells was used for SDS-PAGE at 70 V for 120 min, followed by transfer to a polyvinylidene fluoride (PVDF) membrane and incubation with 5% skim milk at room temperature for 1.5 h. The membrane was then incubated with rabbit antibodies (*AQP5* antibody 1:1000, p-EGFR antibody 1:500, EGFR antibody 1:1000, p-ERK1/2 antibody 1:1000, ERK1/2 antibody 1:1000, p-p38 MAPK antibody 1:500, p38 MAPK antibody 1:500, and GAPDH antibody 1:2000) for 2 h, followed by reservation overnight at 4°C. All the antibodies were purchased from Abcam Inc., Cambridge, MA, USA. Then the membrane was washed three times with tris-buffered saline tween (TBST) (pH 7.4), and goat anti-rabbit IgG labeled with horseradish peroxidase (1:2000) was added, followed by incubation at room temperature for 1 h. Photos were scanned after development with chemiluminescence reagent ECL (Vazyme Biotech Co., Ltd, Jiangsu, China), and Image J software was used to analyze relative protein expression based on gray levels.

### Statistical analysis

SPSS 21.0 software (SPSS Inc., Chicago, IL, USA) was used for statistical analysis. The measurement data were presented as mean ± standard deviation, *t* test was used for comparison between two groups, comparison of multiple groups was conducted with One-way analysis of variance (ANOVA) and pairwise comparison among groups was done with Student-Newman-Keuls (SNK) method. Repeated measures analysis of variance was used for comparisons of cell proliferation at different time points, and Wilcoxon's signed-rank test was performed for non-normally distributed data. The enumeration data were expressed as ratio or percentage and analyzed by chi-square test. *P* < 0.05 was considered statistically significant.

## SUPPLEMENTARY MATERIALS FIGURES


