# Supplementary material for: Effects of AQP5 gene silencing on proliferation, migration and apoptosis of human glioma cells through regulating EGFR/ERK/ p38 MAPK signaling pathway
Source: Oncotarget. 2017 Mar 22;8(24):38444–55. doi: 10.18632/oncotarget.16461 (PMC5503544; doi:10.18632/oncotarget.16461)
Supplement: Supplementary file 1 [file oncotarget-08-38444-s001.pdf]

Effects of AQP5 gene silencing on proliferation, migration and apoptosis of human glioma cells through regulating EGFR/ERK/ p38 MAPK signaling pathway

Supplementary Materials

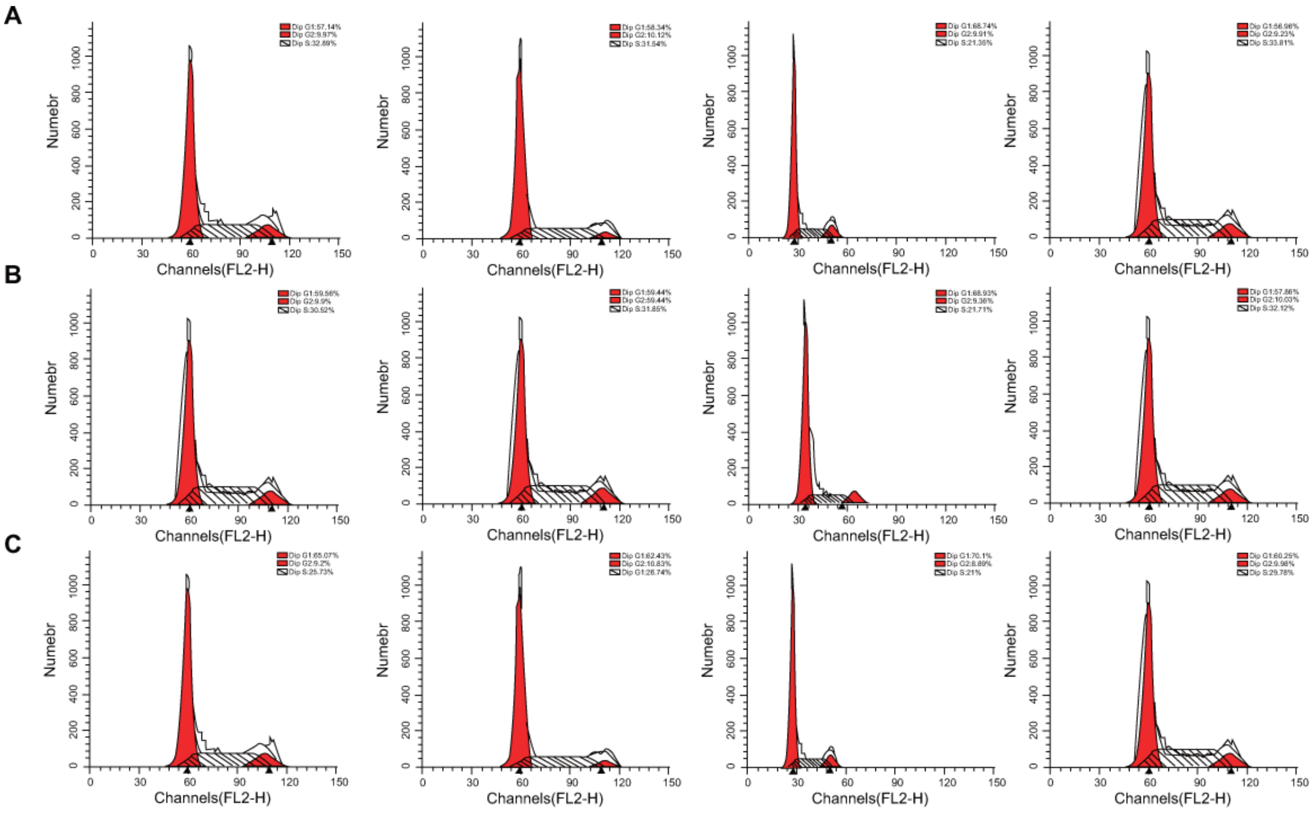

Supplementary Figure 1: Effect of *AQP5* gene silencing on the cell cycle of U87-MG, U251 and LN229 at 24 h after transfection. (**A**) U87-MG; (**B**) U251; (**C**) LN229.
